# Genetic variations in human ATP2B4 gene alter *Plasmodium falciparum* in vitro growth in RBCs from Gambian adults

**DOI:** 10.1186/s12936-022-04359-4

**Published:** 2023-01-05

**Authors:** Fatou Joof, Elena Hartmann, Alison Jarvis, Alhassan Colley, James H. Cross, Marion Avril, Andrew M. Prentice, Carla Cerami

**Affiliations:** 1 Medical Research Council Unit The Gambia at London School of Hygiene and Tropical Medicine, Banjul, The Gambia; 2 Manchester University, Manchester, UK; 3 MalarVx, Inc, Seattle, WA USA

## Abstract

**Background:**

Polymorphisms in *ATP2B4* coding for PMCA4b, the primary regulator of erythrocyte calcium concentration, have been shown by GWAS and cross-sectional studies to protect against severe malaria but the mechanism remains unknown.

**Methods:**

Using a recall-by-genotype design, we investigated the impact of a common haplotype variant in *ATP2B4* using in vitro assays that model erythrocyte stage malaria pathogenesis. Ninety-six donors representing homozygotes (carriers of the minor alleles, T/T (variant), heterozygote T/C and wildtype C/C (ancestral)) carriers of the tagging SNP rs1541252 were selected from a cohort of over 12,000 participants in the Keneba Biobank.

**Results:**

Red blood cells (RBCs) from homozygotes showed reduced PMCA4b protein expression (mean fluorescence intensities (MFI = 2428 ± 124, 3544 ± 159 and 4261 ± 283], for homozygotes, heterozygotes and wildtypes respectively, p < 0.0001) and slower rates of calcium expulsion (calcium t_½_ ± SD = 4.7 ± 0.5, 1.8 ± 0.3 and 1.9 ± 0.4 min, p < 0.0001). Growth of a *Plasmodium falciparum* laboratory strain (FCR3) and two Gambian field isolates was decreased in RBCs from homozygotes compared to heterozygotes and wildtypes (p < 0.01). Genotype group did not affect parasite adhesion in vitro or *var*-gene expression in malaria-infected RBCs. Parasite growth was inhibited by a known inhibitor of PMCA4b, aurintricarboxylic acid (IC_50_ = 122uM CI: 110–134) confirming its sensitivity to calcium channel blockade.

**Conclusion:**

The data support the hypothesis that this *ATP2B4* genotype, common in The Gambia and other malaria-endemic areas, protects against severe malaria through the suppression of parasitaemia during an infection. Reduction in parasite density plays a pivotal role in disease outcome by minimizing all aspects of malaria pathogenesis. Follow up studies are needed to further elucidate the mechanism of protection and to determine if this *ATP2B4* genotype carries a fitness cost or increases susceptibility to other human disease.

## Background

Globally, *Plasmodium falciparum* malaria remains one of the most common infectious diseases, infecting about 241 million people worldwide in 2020 [1]. The majority of people infected with malaria recover without developing life-threatening complications and develop short- and mid-term immunity against future infections and serious sequelae [2]. Host genetic factors are estimated to account for one-quarter of the total variability in malaria severity [3]. Recent genome-wide association (GWAS) and cross-sectional studies have confirmed the protection afforded by known traits such as haemoglobinopathies, the ABO blood group system, and glucose-6-phosphate dehydrogenase (G6PD) deficiency, and have identified new polymorphisms associated with resistance to severe malaria [4–7]. Polymorphisms with some of the strongest associations are in the *ATP2B4* gene and the glycophorin gene cluster. Polymorphisms on the glycophorins appear to be spatially limited to Eastern Africa [6–8]. *ATP2B4* polymorphisms are widely distributed in malaria endemic regions and have been reproducibly linked to altered susceptibility to malaria in multiple African populations [9–11].

*ATP2B4* codes for a plasma calcium membrane ATPase 4 (PMCA4) which is part of the plasma membrane calcium ATPase (PMCA) pump family. PMCAs are ATP dependent pumps that actively remove calcium from the cytoplasm [12]. *ATP2B4* codes for PMCA4b, the primary regulator of RBC calcium concentration. PMCAs are found ubiquitously in eukaryotic cells and are responsible for the expulsion of calcium from the cytosol [13]. There are four isoforms of the PMCAs: PMCA1, PMCA2, PMCA3 and PMCA4. Each of these isoform transcripts can undergo alternative splicing. PMCA4b is a splice variant of PMCA4, expressed in RBCs and coded by *ATP2B4 *[13]*.* PMCA4b is not specific to the RBC, it is ubiquitously expressed in all other cell types tested to date.

**Fig. 1:**
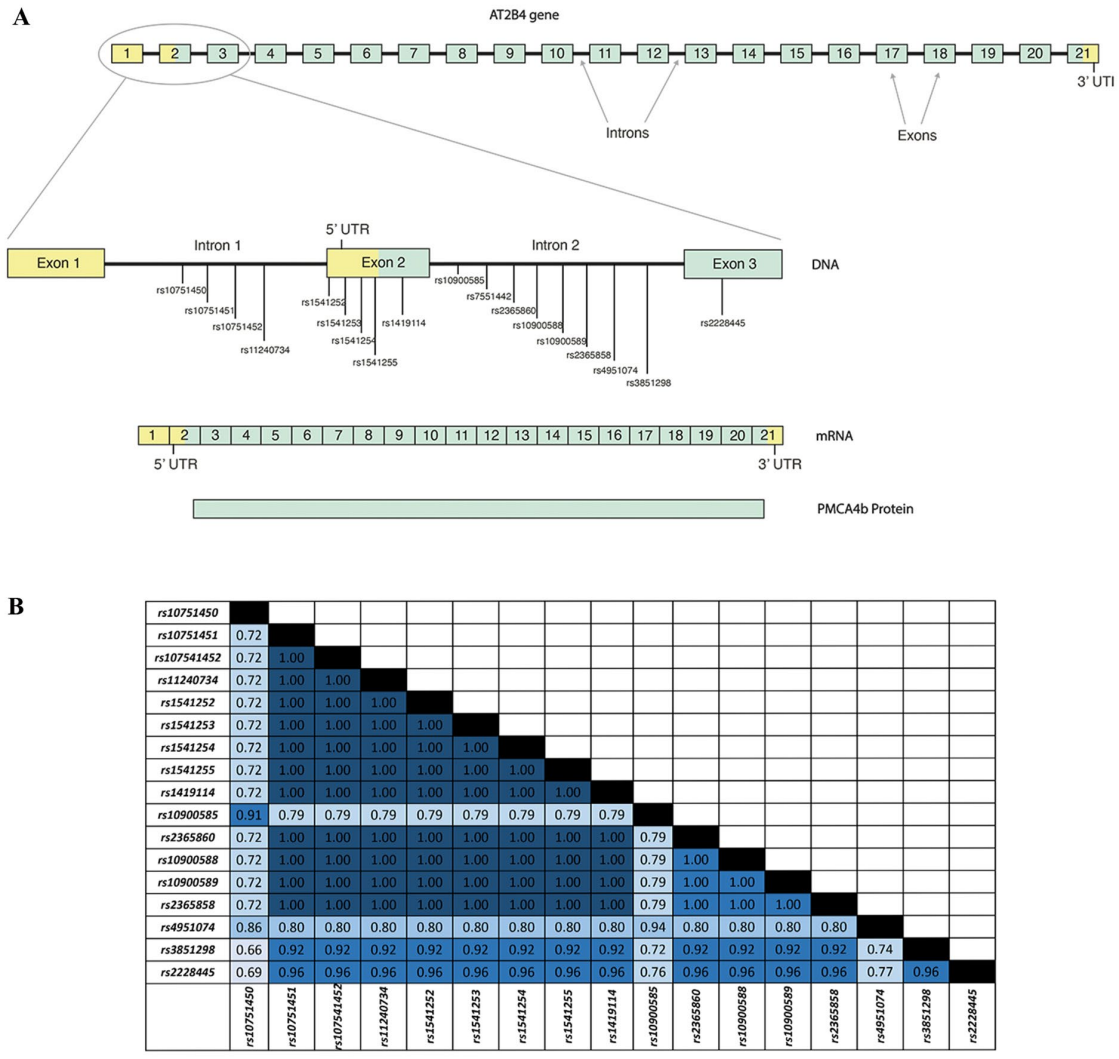
**A** Relative positions of SNPs on *ATP2B4* gene**.** Schematic view of the *ATP2B4* gene, mRNA and protein showing the genomic location of all SNPs associated with malaria altered susceptibility and one SNP (rs7551442) associated with altered erythrocytic MCHC. Boxes represent coding region (exons). Boxes in yellow represent the exons that form the 5’ and 3’ untranslated regions. Boxes in green represent the exons that code for the translated protein. **B** Linkage disequilibrium analysis of *ATP2B4* SNPs associated with malaria protection and PMCA4b function. LD analysis was done using LDlink on the 1000 genome data of Gambian population. Matrix was created to describe the relationship between the SNPs as a function of R^2^ presented. R^2^ between each pair of SNPs are color coded according to strength of the LD (R^2^ = 1 to 0.9, dark blue or ‘strong’; R^2^ = 0.8 to 0.7, medium blue or ‘moderate’ and R^2^ = 0.6, light blue for ‘weak’

The variant haplotype in the *ATP2B4* gene described here will likely impact only RBCs and not the expression of PMCA4b in other cell types, since several of the polymorphisms described are located in the erythroid enhancer region of the gene (Fig. 1).

Previous work has found decreased expression and function of PMCA4b in erythrocytes from individuals with ATP2B4 polymorphisms [14]. Deletion of ATP2B4 alters RBC homeostasis which presents a hostile environment for the parasite [15].

This study is the first time the effect of ATP2B4 polymorphisms on erythrocytes and on malaria pathogenesis from people living in a malaria endemic region has been investigated Here the effects of three common *ATP2B4* genotype groups defined by the tagging SNP rs1541252 on in vitro* P. falciparum* growth, adhesion, invasion are reported.

## Methods

### Study population: Kiang West Longitudinal Population Study (KWLPS) cohort and the Keneba Biobank

The Keneba Biobank contains samples from over 12,000 individuals. The Keneba Biobank, in conjunction with the Keneba Electronic Medical Records System and Kiang West Demographic Surveillance System, forms a substantial research platform, which supports health care provision and research within the Kiang West Longitudinal Population Study [16]. The Keneba Biobank also contains a genetic database which is currently comprised of Illumina HumanExome array data on 80,000 directly genotyped putative functional variants from over 3000 participants. The KWDSS forms the sampling framework for all data collection in this study.

### Ethics statement

The study proposal was reviewed and approved by the Scientific Coordinating Committee of the Medical Research Council Unit The Gambia at London School of Hygiene and Tropical Medicine (MRCG at LSHTM) and ethical approval was granted by the Joint Gambia Government/Medical Research Council Ethics committee (SCC number 1421). The study was conducted according to Good Clinical Practice standards. The study procedures were explained to the donors orally and in writing. Individuals donated 5 mls of whole blood to the study only after the written informed consent was provided.

### DNA extraction

200 uL of the donated blood was aliquoted for DNA extraction to confirm the genotypes obtained from their Keneba Biobank DNA. DNA was extracted using the QIAamp DNA mini kit (Qiagen cat. 51,306) as per manufacturer’s instructions.

### Taqman genotyping

Taqman assay was used to determine SNPs from DNA samples for targets rs2228445, rs2365858, rs54951074 and rs1541252. Taqman SNP assay mix (ThermoFisher Scientific cat. 4,351,379) was used for each target and assay was carried out as per manufacturer’s instructions.

### PMCA4b protein expression

A previously described method with few modifications was used [14]. In brief, erythrocytes were permeabilized with paraformaldehyde to create ‘ghost’ erythrocytes which will allow measurement of PMCA4b expression. To distinguish RBC ‘ghost’ from debris, this was incubated with a lectin, Wheat Germ Agglutinin Alexa Fluor 647 conjugate (Life Technologies) at 1.6 µg/mL. This was simultaneously incubated with a primary antibody that binds to the PMCA4b protein on the membrane of the erythrocyte ‘ghosts’ followed by a secondary fluorescent antibody. Primary antibody, Anti-PMCA4b clone JA3 (Merck Millipore cat.) concentration was optimized at 4 µg/mL and the secondary antibody, Alexa Fluor 488 goat labelled anti-mouse (H + L) (Life Technologies) at 10 µg/mL. For each sample, a negative test was prepared without the primary antibody to assess the background stain of Alexa Fluor 488 on erythrocytes and to control for a failed run. Fluorescence was then measured with BD Accuri™ C6 Plus flow cytometer (BD Biosciences) and only WGA expressing cells (FL4 channel) were gated for PMCA4b expression which was assessed by mean fluorescent intensity (MFI) on (FL1 channel). Flow data were analysed with on FlowJo^®^ (Version 10.1) software.

### Calcium extrusion assay

The activity of the PMCA4b was assessed as previously described [14, 17]. In brief, RBCs were initially loaded with Ca^2+^ using an ionophore, Ionomycin (Molecular Probes/Invitrogen cat. 12,422) in a calcium rich buffer. Calcium loss from the cells was then measured by detecting emitted fluorescence from Fluo-4-AM (Molecular Probes/Invitrogen cat. F14201), a calcium dye indicator that fluoresces when bound to calcium. The dynamics of calcium loss from the RBCs was assessed continuously for 15 min using BD Accuri™ C6 Plus flow cytometer (BD Biosciences).

### *Plasmodium falciparum* culture lines and maintenance

*Plasmodium falciparum* strain FCR3-FMG (MR4, MRA-736) strain was used for most of the in vitro malaria assays. Parasites were maintained under standard conditions using RBCs from healthy donors. Schizonts stage infected RBCs were purified using a magnetic-activated cell sorting system (MACS, Miltenyi Biotec, Inc) for all assays. Parasite strains 952 and 998 were isolated from patients presenting with symptomatic malaria infections at the outpatient clinic at MRC Fajara. Isolates were collected as part of a larger study during the annual malaria transmission seasons (September–January) from 2005 to 2011, as described in [18]. FCR3- VAR2CSA parasite isolates were gifted by Benoit Gamain and colleagues from the Institute National de la Santé et de la Recherche Médicale (INSERM).

#### *Plasmodium falciparum* growth assay

*Plasmodium falciparum* growth assay was carried out as previously described [19]. In brief, MACS purified schizonts were added to the WBC depleted erythrocytes at 1% parasitaemia and 2% haematocrit, plated in triplicates and incubated at 37 °C with 5% CO_2_, 5% O_2_ and 90% N. Parasitaemia at culture initiation (0 h) and after 72 h of culture was confirmed using a BD Accuri™ C6 flow cytometer as described [19]. Parasite growth rates were determined using formula: (final parasitaemia—initial parasitaemia)/ initial parasitaemia.

### Barcoded invasion assay

This was performed as described previously [19]. Donor RBCS were stained with three different concentrations of CellTrace Far Red DDAO dye (Life technologies cat. C34553): 1 uM, 0.5 uM and 0.1 uM. Erythrocytes were then pooled and seeded with MACS purified schizonts at 1% parasitaemia and incubated for 24 h. Parasitaemia was then assessed by flow cytometry as above.

### RT-qPCR for var gene transcripts

500 ul pellet of each parasite culture was dissolved in Trizol (Life Technologies). RNA was extracted using phenol–chloroform and with RNeasy mini extraction kit (Qiagen) as described in [20]. Quantification of var transcripts was done as previously described [21] with the following minor modifications: cDNA was synthesized using Multiscribe reverse transcription kits as per manufacturer’s instructions (Invitrogen). qPCR was done on CFX96 Touch Real-Time PCR detection system (BIO-RAD) using SYBR green supermix (BIO-RAD). The *var* gene transcription profiles were performed using the same set of primers, targeting the IT4/FCR-3 var repertoire as previously described [21].

### *Plasmodium falciparum* iRBC chondroitin sulphate spot binding

*Plasmodium falciparum* FCR3-CSA strain infected RBCs were used in binding assays as previously described [22]. In brief, ‘knobs’ expressing infected erythrocytes were bound on spots of coated Chondroitin Sulphate A Sodium salt (CSA) from bovine trachea (Millipore Sigma) at 100 ug/mL on petri dish (FALCON). Infected erythrocytes per mm^2^ was calculated using the formula: Total counted infected erythrocytes in 4 fields/ 0.1735 (area field 40X).

### Aurintricarboxylic acid (ATA) inhibition assay

*Plasmodium falciparum* iRBC*’s* susceptibility to ATA (Sigma Aldrich cat. A1895-5G) was tested using conventional IC50 drug assay as described in [23]. Experiments were carried out in duplicate for three biological repeats. Parasite’s response to ATA was determined using a nonlinear regression dose–response analysis (with ATA concentration in logarithm) to obtain IC50 value.

### Statistical analysis

Minor Allelic Frequency (MAF) in the population of 175 was determined from the PCR genotyping and LD analysis of the PCR genotypes was conducted in R (package “Genetics”) and LDlink (NIH, NCBI). All results obtained from the flow cytometer (BD Accuri C6) were analysed with FlowJo® software (version 10.1) to determine MFI and parasitaemia. Data analysis and figure generation were carried out using GraphPad Prism (version 9). ANOVA and Dunn’s multiple comparisons were performed to compare the calcium half-lives values, protein expression MFI, growth rate, percentage invasion of wild type, heterozygote and mutant samples obtained. In all analyses, a p-value < 0.05 was considered significant.

## Results

### *ATP2B4* SNPs associated with resistance to severe malaria are in strong linkage disequilibrium (LD)

*ATP2B4* SNPs associated with altered resistance to malaria by GWAS are numerous and found in the region from the first to the third exon spanning a 16.5 kilobase (KB) region (Fig. 1A). Most of the *ATP2B4* SNPs are found in non-coding (intronic) and regulatory regions [e.g., the 5’ untranslated region (5’UTR)] of the *ATP2B4* gene]. Previous analysis of four of these SNPs (rs1541252, rs1541253, ra1419114 and rs2228445) demonstrated that they were in complete LD [13]. To extend this observation, an LD analysis for all 17 SNPs in *ATP2B4* previously linked to malaria susceptibility using LDLink [24] and 1000 genome data on Gambians in Western Division (GWD) was conducted. Overall, a strong pairwise linkage between all SNPs with R^2^ range of 0.66 to 1 was found and characterized the haplotype block (Fig. 1B).

### Recall-by-genotype recruitment

**Fig. 2:**
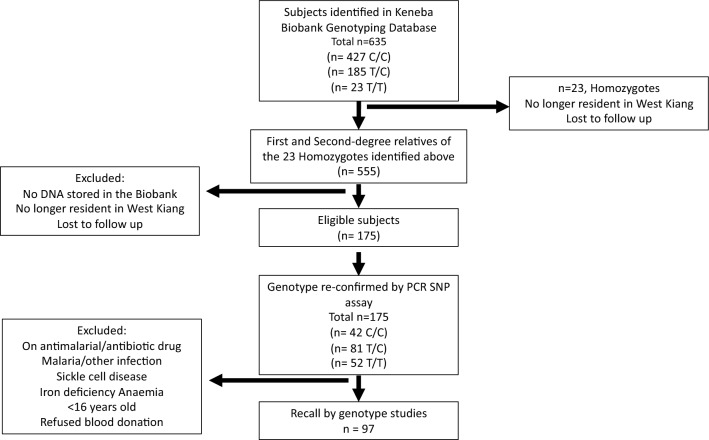
RBC donor identification and selection. Flow diagram

Using a Recall-By-Genotype design, RBC donors were selected from a pre-genotyped cohort of 635 individuals in the Keneba Biobank (MRCG at LSHTM) [16]. Among those, 23 individuals who were homozygous carriers of the minor rs1541252 allele (T/T), were lost to follow up due to migration out of the study area. Nevertheless, 175 ungenotyped first- and second-degree relatives of these 23 donors that met study inclusion criteria were identified and typed for rs1541252 by Taqman PCR. Individuals were further screened and excluded if they no longer lived in the study area or had any of the following: haemoglobin S (heterozygous and homozygous), haemoglobin < 11 g/dL, history of malaria in the past 3 months, known history of any chronic illness or current use of anti-malarials or antibiotics. A total of 96 RBC donors were recruited based on three genotype groups (31 homozygotes for the minor allele (T/T), 35 heterozygotes (T/C) and 30 wildtypes (C/C)) (Fig. 2).

### PMCA4b protein expression in RBCs from donors with *ATP2B4* variant genotype

**Fig. 3:**
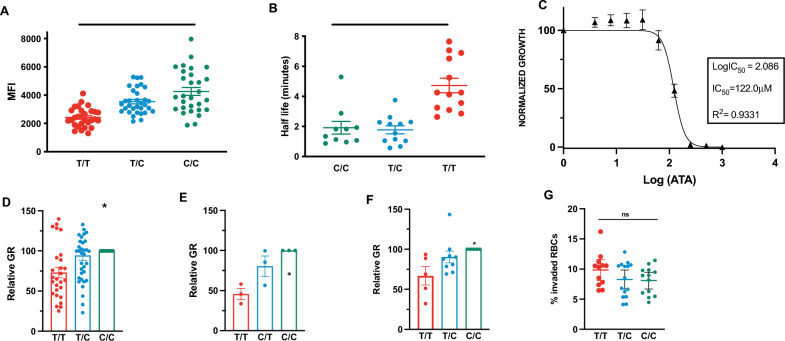
Role of variant *ATP2B4* genotype group on PMCA4b expression, function and *P. falciparum* erythrocytic stage growth. Individuals from homozygous (T/T) genotype (red), heterozygous (T/C) genotype (blue) and wild type (C/C) genotype (green) for the *ATP2B4* variant haplotype **A** Expression of PMCA4b protein in RBCs from homozygous (T/T) donors (n = 29) compared to heterozygous (T/C) donors (n = 29) and wild type (C/C) donors (n = 30). ANOVA followed by Dunn’s multiple comparison testing was used (**** = p > 0.001). **B** Comparison of calcium expulsion from RBCs indicated by calcium half-life in RBCs from homozygous (T/T), (n = 13), heterozygous (T/C) donors (n = 13) and wild type (C/C) donors (n = 13). ANOVA followed by Dunn’s multiple comparison testing was used (**** = p > 0.001). **C** Inhibition of parasite growth by PMCA4b inhibitor aurintricarboxylic acid (ATA) in RBCs (n = 3 from wild type (C/C) donors). Inhibitory concentration at 50% (IC_50_) was calculated using a non-linear regression dose–response model. **D** Growth of *P. falciparum* strain FCR3-FMG in vitro in RBCs from homozygous (T/T) donors (n = 27), heterozygous (T/C) donors (n = 35) and wild type (C/C) (n = 30) **E** Growth of *P. falciparum* field isolates in vitro in RBCs from homozygous (T/T) donors (n = 3), heterozygous (T/C) donors (n = 3) and wild type (C/C) donors (n = 3) were infected with *P. falciparum* clinical strain 998. **F** Growth of *P. falciparum* field isolates in vitro in RBCs from homozygous (T/T) donors (n = 5), heterozygous (T/C) donors (n = 9) and wild type (C/C) donors (n = 5) were infected with *P. falciparum* clinical strain 952. Values for all panels (D) (E) and (F) are presented relative to growth in RBCs from wild type (C/C) donors ANOVA (*p < 0.5). Each dot represents the mean result of triplicate growth assays from each donor and the error bars represent SEM. **G** *P. falciparum* strain FCR3-FMG invasion in RBCs from homozygous (T/T) donors (n = 12), heterozygous (T/C) donors (n = 15) and wild type (C/C) donors (n = 13), Means were compared using one-way ANOVA (ns)

First, the effect of homozygous carriage of the *ATP2B4* minor allele on the expression of PMCA4b protein in the donor RBCs was investigated. ‘Ghost’ RBCs were stained with an antibody directed against PMCA4b followed by fluorescent secondary antibody to determine the relative levels of protein expression. The mean fluorescent intensity (MFI) was compared between genotype groups (Fig. 3A). RBCs from homozygote carriers had significantly decreased protein expression (MFI ± SD, 2427.7 ± 124) compared to RBCs from heterozygotes (3544.4 ± 158.7) and wildtypes (4261.3 ± 283) (p < 0.0001 ANOVA). The difference in protein expression between RBCs from homozygotes versus wildtypes was significant (p < 0.0001, Dunn’s multiple comparison test), but not between RBCs from the heterozygotes and wildtypes (p > 0.05, Dunn’s multiple comparison test).

### Rate of calcium expulsion from RBCs with *ATP2B4* variant genotype

RBCs from donors in all three genotype groups were incubated with fluorescently-labelled Ca^2+^ to assess the efflux efficiency of the PMCA4b using flow cytometry. An exponential decay curve model was used to determine calcium half-life in the RBCs. Figure 3B shows a difference in Ca^2+^ half-life between RBCs from homozygotes compared to those from heterozygotes and wildtypes. The median half-life (4.7 ± 0.5 min) for Ca^2+^ efflux was significantly longer in donor RBCs from homozygote than in RBCs from heterozygote (1.8 ± 0.3 min) and wildtype (1.9 ± 0.4 min) donors (p < 0.0001, ANOVA). Calcium extrusion rates did not differ between wild type and heterozygote individuals (p > 0.05, Dunn’s multiple comparison test).

### *Plasmodium falciparum* growth in RBCs from individuals with the *ATP2B4* variant genotype

To assess whether the PMCA4b could influence *P. falciparum* growth, we incubated the RBCs from wildtype donors with a specific PMCA4b inhibitor aurintricarboxylic acid (ATA) (Sigma Aldrich cat. A1895-5G)) [25]. ATA inhibited the iRBCs growth (Fig. 3C) at an IC_50_ of 112.2 (SD ± 14.2 mM), confirming the crucial role of PMCA4b in *P. falciparum* growth.

In addition, the intraerythrocytic growth of *P. falciparum* (laboratory strain FCR3-FMG) was investigated in donated RBCs from all three genotype groups. A decrease in the parasite growth rate (GR) in RBCs from donors in homozygotes was found compared to heterozygotes and wildtypes (p < 0.05 ANOVA) (Fig. 3D). Growth rates of parasites in RBCs from heterozygotes and wildtypes did not differ (p > 0.05; Dunn’s multiple comparisons). To verify that this phenotype was not an artefact of the laboratory adaptation of the FCR3-FMG strain, in vitro growth of two Gambian clinical isolates of *P falciparum* (isolates 998 and 952) was assessed. These isolates were collected as part of a larger study during the annual malaria transmission seasons (September–January) from 2005 to 2011, as described elsewhere [18]. Both field isolates, 998 (Fig. 3E) and 952 (Fig. 3F) also showed decreased growth rates (GR ± SEM, 45.8% ± 7.01 and 66.9% ± 7.42) in RBCs from homozygous carriers of the minor allele.

### *Plasmodium falciparum *merozoite invasion of RBCs from individuals with the *ATP2B4* variant genotype

Using a barcoded RBC flow cytometry, assay invasion by merozoites in RBCs from individuals with the three *ATP2B4* haplotypes was assessed. The mean percentage of invaded RBCs did not differ between the RBCs from homozygous (9.9 ± 0.8%), heterozygous (8.3 ± 0.7%) or wild-type individuals (8.1 ± 0.6%) (p > 0.05, ANOVA) (Fig. 3G).

### Influence of *ATP2B4* variant haplotype on parasite adhesion in vitro

**Fig. 4:**
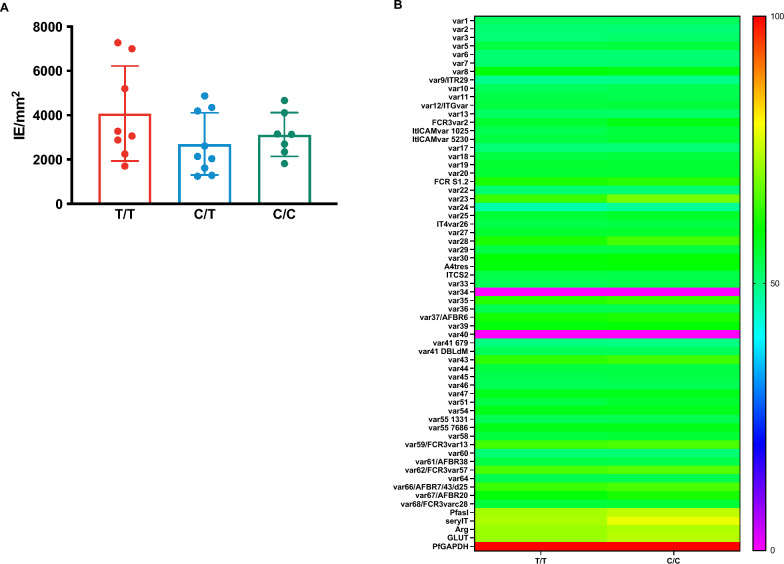
Role of variant *ATP2B4* genotype group in cytoadherence and var gene expression of parasitized RBCs. Donor RBCs were infected with a *P. falciparum* strain expressing var2CSA (FCR3-var2CSA). The number of infected RBC binding to CSA per mm2 was quantified for RBCs from individuals in homozygous (T/T) donors (n = 8), heterozygous (T/C) donors (n = 11) and wild type (C/C) donors (n = 8). Adhesion was measured and compared between the three haplotypes using one-way ANOVA, ns). **B** RNA was extracted from donor RBCs infected with *P. falciparum* strain FCR3-var2CSA. RT-PCR was conducted to detect four variants of *var2CSA* which are known to be expressed in this parasite line. Heat map showing the relative expression profile of the entire *var gene* repertoire (n = 63) excluding var2CSA of FCR3 strain. Parasites were grown in homozygous (T/T) donors (n = 8) and wild type (C/C) donors (n = 8) RBCs and RT-PCR performed following RNA extraction. No significant difference in the expression of any of the *var* genes tested between parasites grown in the homozygous (T/T) and wild type (C/C) donors infected RBCs

Finally, the adhesive properties of malaria-infected RBCs from donors with the three different genotypes was examined using two approaches. First, an in vitro system designed to mimic the adhesion of malaria-infected RBCs to the placenta was used. RBCs were infected with a *P. falciparum* line expressing *VAR2CSA (FCR3-VAR2CSA),* a gene that codes for a parasite ligand known to bind chondroitin sulphate (CSA) expressed in the placenta. The *var2CSA* system was chosen because of the relative ease of the assay and our ability to perform it under field conditions. Furthermore, VAR2CSA is the only *var* gene product known to bind CSA and it is unusually well conserved between parasite isolates [21, 26] The number of parasite-infected RBCs bound per mm^2^ of a CSA-coated plastic surface was assessed. No differences were observed in the binding of parasite-infected RBCs from the homozygous donors (infected RBCs /mm^2^ ± SD, 4079 ± 756) heterozygous (2704 ± 545) or wildtypes (3130 ± 373) (p > 0.05, ANOVA) (Fig. 4A). Second, the expression of other *var* genes using RT-PCR in parasites grown in the RBCs from participants in homozygotes and wildtypes was assessed. No difference in expression of any of the *var* genes tested were detected (Fig. 4B).

## Discussion

The strength of the study presented here includes the parsimonious recruitment of participants by means of the recall by genotype design and the existence of a field laboratory permitting assays on freshly drawn blood. We present evidence that a variant haplotype of *ATP2B4,* composed of 17 SNPs in tight LD and represented here by the tagging SNP rs1541252, decreases PMCA4b protein expression and its function of calcium expulsion and affects *P. falciparum* growth in RBCs. The variant *ATP2B4* genotype did not affect iRBC adhesion to the host receptor CSA, nor did it alter *var* gene expression. Decrease of the growth of the *P. falciparum* FCR3-FMG laboratory strain in RBCs from homozygous subjects were also confirmed in clinical isolates from The Gambia. The entire study was performed in a field laboratory in rural Gambia using freshly collected RBCs and as such, has multiple limitations. Most importantly, attempts to directly measure intracellular calcium in the RBCs were unsucessful. In addition, mRNA for PMCA4b was not measured. There was neither a fluorescent microscope at the field station nor a camera attached to the AccuriC6 flow cytometer, so visualization of the protein expression and localization of PMCA4b on the RBC membranes was not done.

The observations on the relationship between several polymorphisms in *ATP2B4* and risk of malaria was first demonstrated in Ghanaian and Gambian populations [4] and was subsequently replicated in other African [7], Asian and Oceanic populations [9]. The average minor allele frequency is 0.32 in Kenyan [7], 0.4 in Senegalese [27] and 0.36 in all African populations. Recent studies have shown that PMCA4b expression in RBCs is controlled by an erythroid-specific transcription enhancer site and that there is a specific transcript of *ATP2B4* found only in RBCs [10, 14]. All 17 of the SNPS within the *ATP2B4* haplotype variant defined here are near or in this erythroid-specific transcription enhancer (Fig. 1). One of the SNPs (rs10751451) disrupts a GATA motif and the same SNP has previously been shown to associate with *ATP2B4* expression levels and calcium concentration in RBCs [14]. Due to the very strong LD across this region of the ATP2B4 gene, this work is unable to address the possibility that rs10751451 may be the single causal variant in *ATP2B4* that mediates protection from malaria as previously proposed [10]

The normal blood plasma calcium level is 1.8 mM [28], yet RBCs are able to maintain an intracellular calcium concentration between 30 to 60 nM. Excessively high intracellular Ca^2+^ concentrations lead to numerous types of erythrocyte dysfunction [21–23]. Most importantly, Ca^2+^ plays a role in the regulation of other ions, including Na^+^ and K^+.^ An increase in erythrocytic Ca^2+^ concentration activates the Gardos channel which leads to erythrocyte dehydration and shrinkage similar to that observed in sickle cell disease [28, 29].

The source of calcium for post-invasion parasite development in RBCs is not fully understood, but increasing levels of Ca^2+^ are observed as the parasite grows from rings to schizonts and depletion of calcium would result in an arrest of growth [30–32]. Shortly before release of merozoites from the RBCs, Ca^2+^ increases sharply which results in swelling and degradation of the parasitophorous vacuole and the RBC membrane [31–35]. Several calcium-dependent enzymes are involved in this process including, sub-like protease 1 (SUB1), a *Pf*-perforin-like protein and CDPK5 [33, 36, 37]. Additional roles of calcium during the RBCs invasion have also been established. It is however still unclear whether the Ca^2+^ origin is exclusively extracellular or has an intracellular erythrocytic source as well [38].

There are two possible models which could explain the mechanism by which the alterations in PMCA4b function could protect against severe falciparum malaria.

**Fig. 5:**
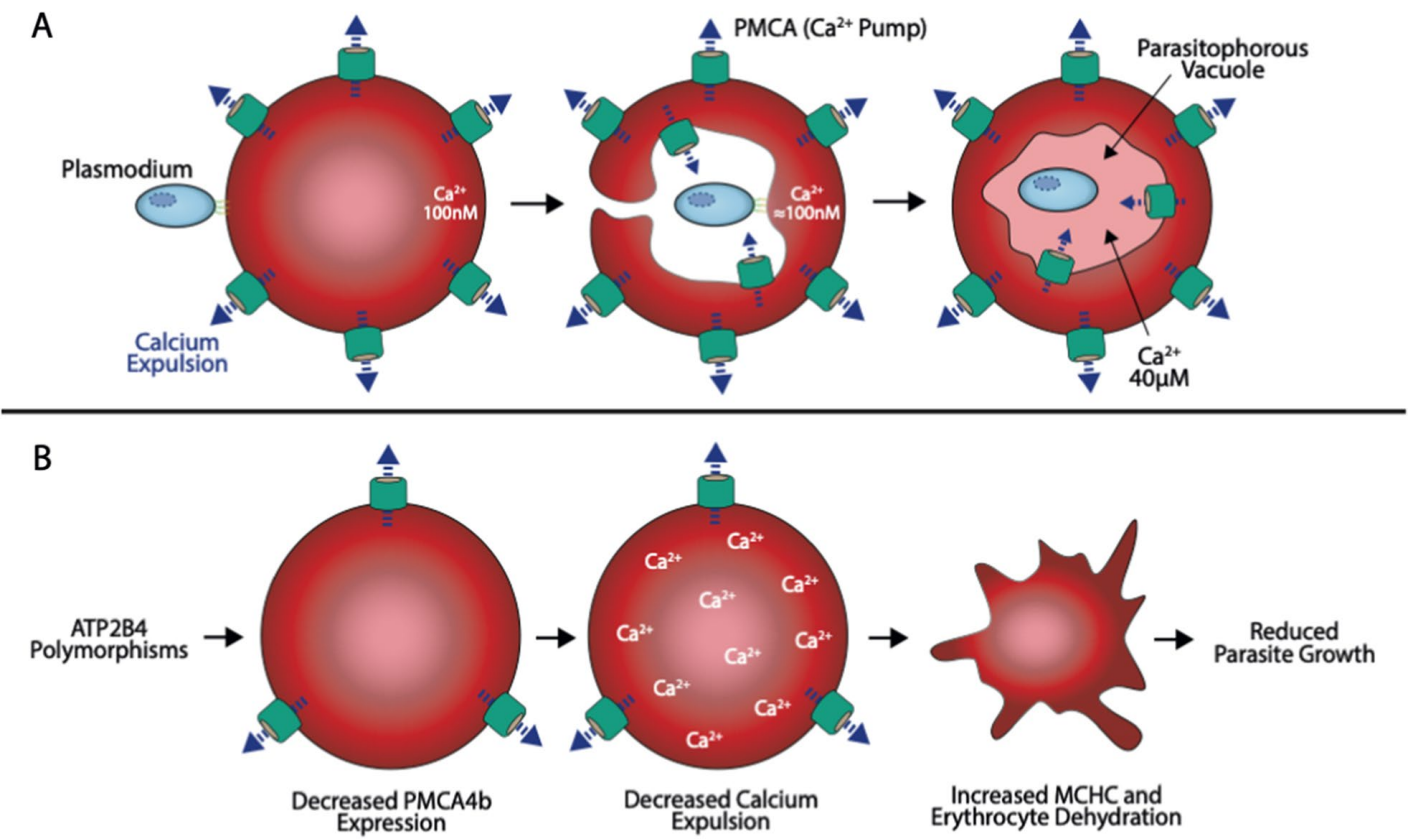
A proposed model of how *ATP2B4* variant haplotype could protect from malaria: **A** Illustration shows invagination of erythrocyte membrane during invasion and formation of the parasitized vacuole with the inverted PMCA4b. **B** A second proposed model of how *ATP2B4* variant haplotype may protect from malaria: RBCs with variant *ATP2B4* variant haplotype will have increased concentration of Ca^2+^ resulting to dehydration

Model 1: *Malaria parasite is starved of calcium*: During the invasion of the merozoite into the RBC, there is an invagination of the erythrocyte plasma membrane such that the usually exterior RBC membrane, along with its receptors and transporters, faces into the parasitophorous vacuole (PV) and supplies the parasites’ calcium needs (Fig. 5A). Decreased calcium in the parasitophorous vacuole would decrease parasite viability.

Model 2 (proposed by Lessard et al. [15]): *RBCs are dehydrated and at increased risk of lysis*: The impaired PMCA4b expression and activity results in increased intraerythrocytic Ca^2+^ concentration which disrupts ion homeostasis of the RBC and results in its dehydration. Dehydration causes the cell to shrink and leads to premature lysis and clearance of malaria infected RBCs [28] (Fig. 5B). Either or a combination of these pathways might be responsible for the impairment of *P. falciparum* growth that we observed in RBCs with *ATP2B4* variant haplotype.

The finding that *P. falciparum* growth in RBCs from homozygotes for the ATP2B4 variant, with decreased levels of PMCA4b is inconsistent with a recent publication by Villegas-Mendez et al. [39]. These authors show that if the *ATP2B4* gene is ablated in a mouse model, there is no difference in the growth of *Plasmodium berghei, Plasmodium yoelii* or *Plasmodium chabaudi* following an in vivo malaria infection between wildtype mice and those without the ablated ATP2B4 gene. These finding presented here are consistent, however with a recently human study on *P. falciparum* published [40] that showed a decrease in parasite densities in malaria infected children with the protective ATP2B4 gene polymorphisms.

A key aim of malaria GWAS studies is to identify possible new targets for anti-malarial drug development [41]. As part of this effort and to provide proof of principle, we tested the PMCA4b inhibitor, aurintricarboxylic acid (ATA), which is under investigation as a blood pressure modulator [42]. These experiments reveal that the IC_50_ value for parasite inhibition was over 100 × higher than common drugs such as chloroquine and artemisinin*.* Because of PMCA4b’s central role in calcium homeostasis in multiple cell types [43–46], future work could be focused on the identification of an anti-malarial compound to block the erythrocyte-specific enhancer in *ATP2B4* rather than the calcium transporter itself.

## Conclusion

Taken together, these data are consistent with the conclusion that polymorphisms in *ATP2B4* common in African populations and inherited as a large haplotype block, protect against severe malaria by controlling parasite density. Reduction in parasite density plays a pivotal role in disease outcome as it can ameliorate all of the multifaceted complications that lead to severe outcomes (including cerebral malaria, respiratory distress and severe anaemia) [48,49].

Although cytoadherence is critical to some of these complications, host immune response to the presence of the malaria parasite also plays an important role. For example, the reaction against the parasite pigment released during RBC lysis is the cause of the cyclic febrile episodes in malaria disease [49]. Additionally, the malaria pigment and hypoxia can contribute to the activation of cytokines which has a role in cerebral malaria and respiratory distress [26, 50]. Severe anaemia in malaria disease is due to loss of lysed cells by high levels of clearance of infected RBCs by the host immune system correlated with parasite density. Therefore, the reduction of parasite growth mediated by the variant *ATP2B4* haplotype will likely diminish each of these risks and thus explain its association with severe malaria.

## Data Availability

All data will be made available to researchers upon reasonable request to the study PI and clearance by the MRCG Scientific Coordinating and Ethics Committees.

## Abbreviations

ATA: Aurintricarboxylic acid
CSA: Chondroitin sulphate
DFID: UK Department for International Development
GWAS: Genome-wide association study
G6PD deficiency: Glucose-6-phosphate dehydrogenase deficiency
LD: Linkage disequilibrium
MRCG at LSHTM: Medical Research Council Unit The Gambia at London School of Hygiene and Tropical Medicine
MFI: Mean fluorescence intensities
RBCs: Red blood cells
PMCA4: Plasma calcium membrane ATPase 4
PMCA: Plasma membrane calcium ATPase
UK MRC: UK Medical Research Council
