# Correction: Genetic variations in human ATP2B4 gene alter *Plasmodium falciparum* in vitro growth in RBCs from Gambian adults

**DOI:** 10.1186/s12936-023-04565-8

**Published:** 2023-05-25

**Authors:** Fatou Joof, Elena Hartmann, Alison Jarvis, Alhassan Colley, James H. Cross, Marion Avril, Andrew M. Prentice, Carla Cerami

**Affiliations:** 1 Medical Research Council Unit The Gambia at London School of Hygiene and Tropical Medicine, Banjul, The Gambia; 2 Manchester University, Manchester, UK; 3 MalarVx, Inc, Seattle, WA USA

**Correction: Malaria Journal (2023) 22:5** 10.1186/s12936-022-04359-4

Following publication of the original article [1], it came to the authors’ attention that TT had been written instead of CC and vice versa in the following parts of the article: the main text body, the figures legends, and Figs. 3A, 3B, 3D, 3G and 4D. The article has since been updated to correct this. Note that while these changes are important for ensuring that the article can easily be correctly interpreted, they do not change the overall results or conclusions of the article.

The authors thank you for reading this erratum and apologize for any inconvenience caused.
